# Mechanisms of Durability Degradation in Recycled Fine Aggregate Concrete of Varying Strengths Induced by Chloride and Sulfate Dry–Wet Cycles

**DOI:** 10.3390/ma18214985

**Published:** 2025-10-31

**Authors:** Chunhong Chen, Kamara Alimatu Adama, Ronggui Liu, Yunchun Chen, Xiaolin Zhang, Hui Liu

**Affiliations:** 1 Faculty of Civil Engineering and Mechanics, Jiangsu University, Zhenjiang 212013, China; 2 School of Urban Construction, Changzhou University, Changzhou 213164, China; kamarralima@gmail.com (K.A.A.); cloud221@126.com (Y.C.); zhang0708930@163.com (X.Z.);; 3 State Key Laboratory of Silicate Materials for Architectures, Wuhan University of Technology, Wuhan 430070, China

**Keywords:** recycled fine aggregate concrete, dry–wet cycles, corrosive environment, free chlorine ion content, performance degradation

## Abstract

With the increasing demand for sustainable building materials, it is essential to investigate the durability of recycled fine aggregate concrete (RFAC) under corrosive environmental conditions. This study systematically assessed the performance of RFAC with three compressive strengths after dry–wet cycles in chloride and sulfate environments, respectively. The experimental program encompassed measurements of compressive strength, mass variation, porosity, ion penetration depth, and free ion content, complemented by comprehensive microstructural characterization. Results show that under sulfate exposure, 20 MPa and 40 MPa RFAC suffered significant strength losses of 60.1% and 18.0% after 70 cycles, while 60 MPa RFAC gained 2.5% strength. In chloride environments, 20 MPa and 40 MPa RFAC experienced strength reductions of 30.7% and 6.9%, whereas 60 MPa RFAC increased in strength by 6.6%. Compared to sulfate exposure, all groups exhibited slight mass increases or porosity reduction under chloride exposure, with high-strength RFAC showing the most noticeable densification. The chloride penetration depth in RFAC of 60 MPa was measured at 14.65 mm, representing a 41.0% reduction compared to RFAC of 20 MPa; sulfate penetration depth was 17.84 mm, which is 44.6% lower than that of the 20 MPa counterpart. Microstructural analysis revealed that sulfate-induced ettringite and gypsum formation triggered crack propagation, while chloride mainly affected pore structure through crystallization and filling, and the formation of C-S-H in high-strength RFAC inhibits pore expansion and mitigates deterioration.

## 1. Introduction

With the acceleration of global urbanization, concrete has become the most widely used construction material. However, its production and disposal have caused serious problems, including greenhouse gas emissions [1], depletion of natural sand and gravel resources [2], and the accumulation of construction solid waste [3,4,5]. The recycling of construction waste has thus become a critical strategy for mitigating resource scarcity and reducing environmental impacts. Among various recycling approaches, the conversion of demolished concrete into recycled coarse aggregate (RCA) and recycled fine aggregate (RFA) for use in producing recycled aggregate concrete (RAC) represents a high-value utilization pathway. Nevertheless, due to the presence of adhered aged mortar, recycled aggregates generally exhibit lower apparent density, higher porosity, and a weaker interfacial transition zone (ITZ), leading to inferior mechanical performance and durability of RAC compared to natural aggregate concrete [6].

In recent decades, the durability evolution of RAC under the coupled effects of salt attack and dry–wet cycles has attracted considerable research interest. Such environments are prevalent in coastal, saline lake, and industrial regions. Under salt erosion conditions, chloride and sulfate attacks exert the most significant influence on the performance of RAC. Chloride salts primarily trigger reinforcement corrosion and crystallization damage, while sulfates tend to form expansive products such as ettringite and gypsum, causing volume expansion and cracking in concrete. Given the performance characteristics of RAC, in-depth investigation into its deterioration mechanisms under the coupled effects of chloride and sulfate attack along with dry–wet cycles is of significant engineering importance. Researchers have conducted extensive investigations into the mechanical properties, durability, and microstructure of recycled concrete incorporating RCA. Significant efforts have been devoted to improving aggregate quality and enhancing the performance of the interface transition zone (ITZ), with studies proposing the use of mineral admixtures and interfacial modification techniques to improve durability [7,8,9,10]. Furthermore, advancements have been achieved in the graded utilization of recycled aggregates, mix proportion optimization, and the characterization of multi-generation recycled aggregates [11,12,13]. Nevertheless, research on the durability of RFAC under aggressive environmental conditions remains limited. To date, no comparative studies have been reported on the performance of recycled fine aggregate concrete with varying strength levels subjected to coupled chloride and sulfate dry–wet cyclic exposure. The anti-corrosion performance of concrete is primarily governed by its internal microstructural characteristics, particularly the extent and distribution of microcracks. These microcracks are predominantly located in critical regions such as the ITZ between aggregates and cement paste, internal aggregate pores, and capillary pores within the cementitious matrix, collectively serving as preferential pathways for the ingress of corrosive agents [14]. In the case of RAC, the strength grade plays a crucial role in determining resistance to chloride and sulfate attack. Variations in strength grades are fundamentally attributed to differences in water-to-cement ratio and the resulting degree of microstructural densification, both of which directly influence the diffusion rate of aggressive ions and thereby govern the macroscopic deterioration mechanisms. To date, there has been insufficient comparative research on the durability evolution of RAC under dry–wet cyclic conditions in both chloride and sulfate environments, and the intrinsic relationship between strength grade and cyclic durability remains insufficiently understood.

Therefore, this study designed three strength grades of RFAC and investigated their deterioration behavior under coupled chloride and sulfate-induced dry–wet cyclic conditions from a multi-scale perspective. The differences in salt erosion mechanisms were clarified, and the critical role of strength levels in determining durability was revealed. The research framework includes: (i) evaluating the macroscopic performance evolution of RAC20, RAC40, and RAC60 during cycling, including compressive strength, mass loss, and porosity changes; (ii) analyzing durability-related parameters such as chloride and sulfate ion penetration depth and free ion content; and (iii) employing SEM and XRD analyses to reveal crack development and the formation of corrosion products, thereby establishing the relationship between concrete strength level and durability performance. The findings are expected to provide theoretical insights and experimental data to support the rational design and durability evaluation of RFAC. Furthermore, the results contribute to a deeper understanding of RAC service behavior in complex corrosive environments.

## 2. Materials and Methods

### 2.1. Materials and Mixtures

The cementitious materials used in this study were commercially available, including P.O 32.5 cement, P.O 42.5 cement, silica fume, and fly ash. The chemical compositions and basic properties of these binders are provided by the manufacturer, Jiangsu Jinfeng Cement Co., Ltd. (Changzhou, China) as presented in Table 1. A polycarboxylate-based high-performance superplasticizer with a water-reducing efficiency of 25% was employed as the chemical admixture.

**Figure 1:**
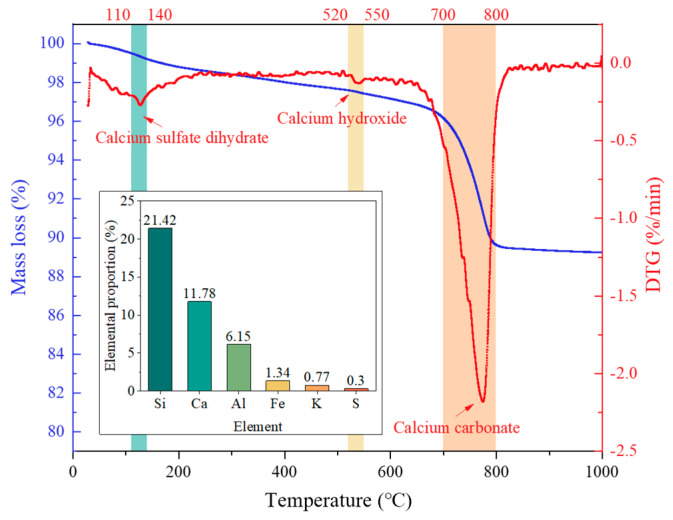
Thermogravimetric and elemental analysis of RFA.

The natural coarse aggregate (NCA) was limestone with a particle size ranging from 4.75 to 20 mm, while the RFA was derived from crushed waste concrete and has a particle size of 0.075 to 4.75 mm. Both aggregates were supplied by a local building materials company, namely Jiangsu Lvhe Environmental Technology Co., Ltd. (Changzhou, China). Their physical and mechanical properties were tested in accordance with the standards of GB/T 14684-2022 [15] and GB/T 14685-2022 [16], and the results are listed in Table 2. Thermal decomposition and elemental composition of the RFA were analyzed, as shown in Figure 1. From Figure 1, the mass percentages of Ca(OH)_2_ and CaCO_3_ were obtained as 3.29 wt% and 12.51 wt%, respectively. Subsequently, these data were used to estimate the amount of hydrated cement by converting the Ca(OH)_2_ content. It was assumed that 25% of the CaCO_3_ originated from the carbonation of Ca(OH)_2_ and 75% of CaCO_3_ is the original filler or residual aggregate. Based on this assumption, the quantity of unhydrated cement in the adhered mortar was calculated relative to the original cement content (30%). Using this methodology, the unset cement content in RFA was determined to be approximately 12%. The particle size distribution and grading curves of both NCA and RFA met the requirements of Chinese standards GB/T 14685-2022 and GB/T 25176-2010 [17].

Concrete mixtures were designed with target compressive strengths of 20, 40, and 60 MPa, denoted as RAC20, RAC40, and RAC60, respectively. The mix proportions were calculated using the absolute volume method (Table 3). Prior to specimen preparation, the RFA was pre-soaked in water for 24 h without adding extra water (i.e., without changing the total water content), a procedure that is known to improve the workability of RAC [18]. In addition, RAC20 was prepared using P.O 32.5 cement, while RAC40 and RAC60 were made with P.O 42.5 cement.

**Figure 2:**
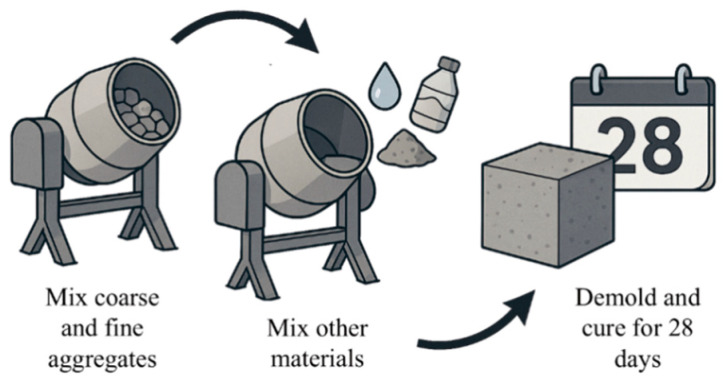
Specimen preparation process.

**Figure 3:**
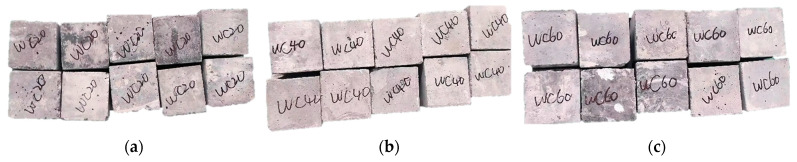
The RAC specimens for curing 28 days: (**a**) RAC20, (**b**) RAC40 and (**c**) RAC60.

According to the Chinese standard GB/T 50081-2019 [19], cube specimens with dimensions of 100 × 100 × 100 mm^3^ were cast. The number of specimens and their specific testing purposes are listed in Table 4. During mixing, coarse and fine aggregates were first blended, followed by the addition of water, cementitious materials, and the superplasticizer, ensuring a homogeneous distribution of all components. After casting, specimens were demolded after 24 h and cured in a standard curing chamber for 28 days, as shown in Figure 2 and Figure 3.

### 2.2. Exposures

**Figure 4:**
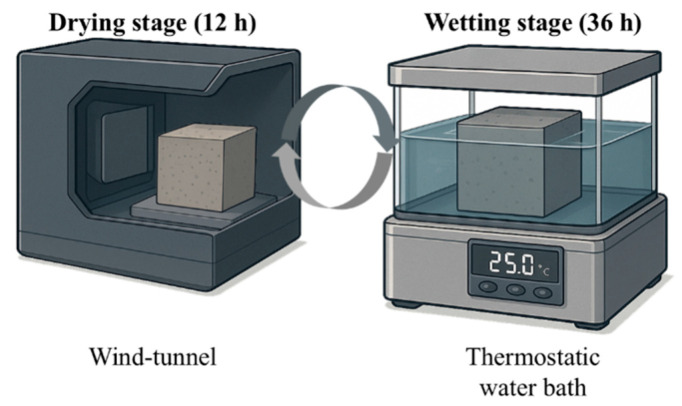
Testing equipment and procedures.

The exposure regime in this study was designed as chloride and sulfate dry–wet cycles, following the American standard ASTM D1141-98 (2021) [20]. As illustrated in Figure 4 the drying stage was carried out in a small-scale wind tunnel laboratory, with an air velocity of 6.0 ± 0.1 m/s, maintaining the specimen surface temperature at 25 °C and the relative humidity at 50%. During the wetting stage, specimens were fully immersed in a thermostatic water bath, with the solution level covering the specimen surface and the water temperature controlled at 25 °C.

The immersion solutions consisted of 10% chloride and 10% sulfate solutions, with the concentrations adopted from Yang et al. [21]. The solutions were regularly monitored and replaced to maintain stability. The duration of the drying stage was 36 h, followed by a 12 h wetting stage, resulting in a complete dry–wet cycle every 48 h. The design of this exposure protocol was based on previous studies [22,23,24].

### 2.3. Test Methods

The physical–mechanical properties of concrete included open porosity and compressive strength, tested in accordance with GB/T 50081-2019. Open porosity was defined as the ratio of open pore volume to the total concrete volume, and was indirectly obtained by measuring the water uptake of saturated specimens. Compressive strength was determined using the testing machine produced by Jinan Huaheng Testing Equipment Co., Ltd. in Shandong, China.

**Figure 5:**
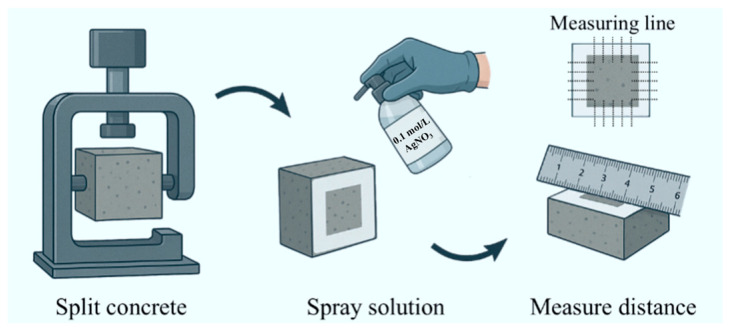
Measurement of chloride ion corrosion depth.

Durability tests included mass variation, penetration depth of aggressive ions, and free ion content at different depths. According to GB/T 50082-2009 [25], mass variation was evaluated by weighing and tracking three specimens per group throughout the exposure cycles. The chloride penetration depth was measured following the procedure shown in Figure 5. Each cubic specimen was split into two halves, and the freshly exposed surface was sprayed with 0.1 mol/L AgNO_3_ solution. After 15 min, the chloride-contaminated area turned white. The distance from the unstained edge to the specimen edge was measured with a steel ruler, and the average value was recorded as the chloride penetration depth. The procedure for sulfate penetration was the same, except that phenolphthalein solution was used, and the boundary between the colored and uncolored zones was measured.

The free chloride content was determined according to ASTM C114-23 (2023) [26]. Concrete powders were sampled at depths of 0–30 mm from the exposed surface at 10 mm intervals. The powders were leached with distilled water, diluted, and titrated using silver nitrate solution with potassium chromate as the indicator, from which the free chloride content was calculated. Free sulfate content was determined using the same sampling and leaching procedure, followed by boiling and washing with dilute hydrochloric acid to remove impurities. Barium chloride solution was then added to precipitate sulfate ions. The precipitates were ignited in a high-temperature furnace, weighed, and used to calculate sulfate content.

After the completion of dry–wet cycling, microstructural analyses were performed. Scanning electron microscopy (SEM) was used to observe the microstructure of concrete specimens, while X-ray diffraction (XRD) was applied to identify corrosion products by comparative analysis.

## 3. Results

### 3.1. Initial Performance Befor Cycling

**Figure 6:**
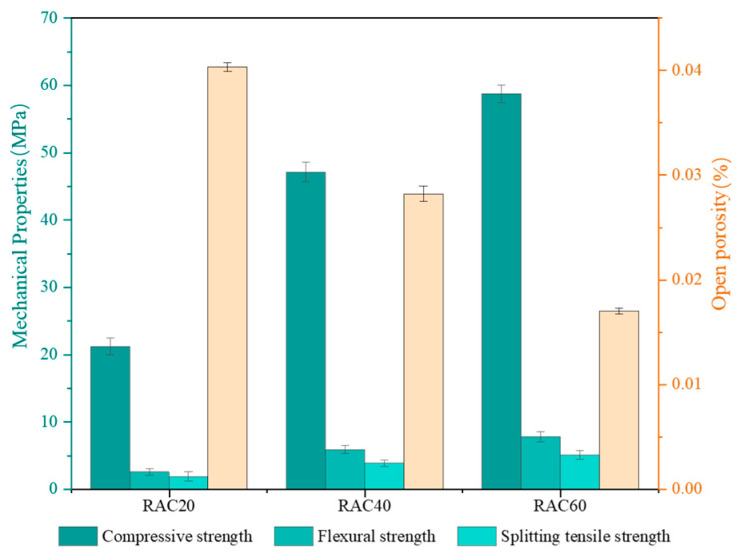
Initial mechanical and porosity properties.

Figure 6 illustrates the pronounced differences in initial mechanical and porosity properties among the three groups of concrete with different strengths, and the specific compressive strength test data can be found in Table A1 of the Appendix A. All mixtures achieved their target compressive strengths, while the flexural and splitting tensile strengths exhibited consistent trends. A clear negative correlation was observed between open porosity and compressive strength. The open porosity decreased from 4.03% in RAC20 to 1.70% in RAC60, representing an overall reduction of approximately 57.8%. This result indicates that higher strength grades of concrete are strongly associated with a denser pore structure, and that the refinement of the matrix porosity provides the direct physical basis for strength enhancement [27,28].

### 3.2. Physical–Mechanical Properties

#### 3.2.1. Compressive Strength

**Figure 7:**
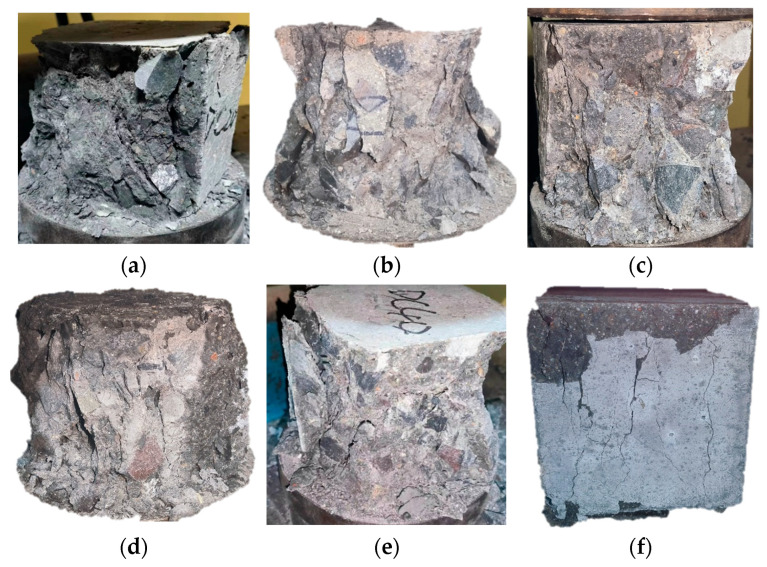
The RAC specimens under 40 dry–wet cycles after the compressive strength test, (**a**,**d**) RAC20, (**b**,**e**) RAC40, (**c**,**f**) RAC60 (**a**–**c**) under the chloride exposure, (**d**–**f**) under the sulfate exposure).

Following the completion of 40 drying–wetting cycles, the specimens were subjected to compressive strength testing. The resulting damage characteristics are presented in Figure 7. As shown in the figure, the low-strength specimens suffered severe damage after being subjected to pressure, with a large amount of concrete peeling off around the specimens, resulting in significant damage to the overall integrity. While the high-strength specimens had a relatively mild damage degree after being subjected to pressure, although there was a small amount of concrete peeling off in some local areas, they still maintained good structural integrity. Moreover, by comparing specimens of the same strength grade, it can be clearly seen that the specimens exposed to sulfate dry–wet cycles had a lower damage degree after being subjected to pressure than those exposed to chloride salt dry–wet cycles.

**Figure 8:**
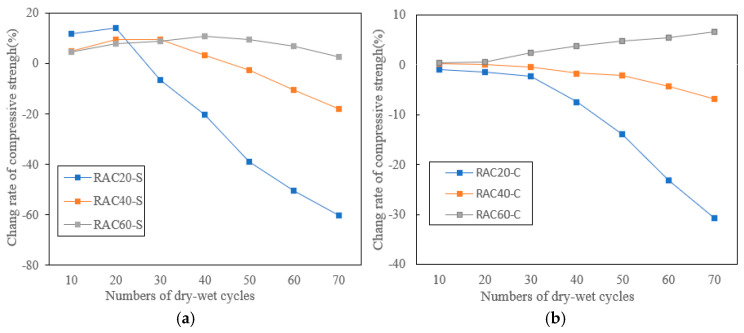
Compressive strength variation in RAC under (**a**) sulfate and (**b**) chloride dry–wet cycles.

Figure 8 presents the compressive strength variation in RACs with different design strengths (RAC20, RAC40, and RAC60) under chloride and sulfate dry–wet cycles, and the specific test data can be found in Table A2 of the Appendix A. Overall, the results indicate that concrete generally exhibited a moderate strength increase during the early stage of exposure (10–20 cycles), followed by progressive deterioration in the mid-to-late stage (≥30 cycles). The degradation rate was strongly dependent on both the design strength and the type of aggressive medium.

As shown in Figure 8a, in the sulfate environment, RAC20 showed an initial strength gain of approximately 14.2% at 20 cycles but experienced rapid deterioration afterward, losing as much as 60.1% of its strength by 70 cycles. RAC40 maintained a 9.6% gain at 30 cycles but subsequently declined, reaching an 18.0% loss at 70 cycles. By contrast, RAC60 demonstrated much greater resistance: its strength remained within the range of +8.9% to +10.7% during 30–50 cycles and still retained a net gain of +2.5% after 70 cycles, suggesting that a denser matrix effectively mitigated both sulfate crystallization pressure and chemical attack [29].

As shown in Figure 8b, in the chloride environment, the deterioration trend was less pronounced. RAC20 exhibited a continuous decrease from the onset, with a 30.7% loss by 70 cycles. RAC40 showed relatively minor fluctuations, with only a 6.9% reduction after 70 cycles. Remarkably, RAC60 exhibited steady strength improvement throughout the exposure, achieving a net gain of +6.6% after 70 cycles. These findings highlight that, under chloride cycling, low-strength concrete suffers rapid deterioration due to higher porosity and a weaker ITZ, while high-strength concretes benefit from a dense matrix and pozzolanic activity, showing superior durability.

The initial strength enhancement observed across all groups can be attributed to pore filling by salt crystallization and secondary pozzolanic reactions, which temporarily densified the microstructure [30]. However, with increasing cycles, the higher porosity and weaker ITZ associated with RFA in RAC20 and RAC40 facilitated the accumulation of corrosion products and crack propagation, accelerating strength loss. In contrast, RAC60, with its low water–binder ratio and higher content of supplementary cementitious materials, developed a refined pore structure that limited ion ingress and maintained mechanical stability.

#### 3.2.2. Mass Change

**Figure 9:**
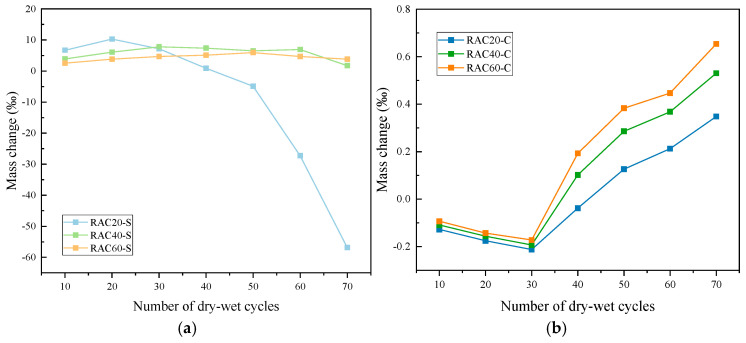
Mass variation in RAC with different strengths under (**a**) sulfate and (**b**) chloride dry–wet cycles.

Figure 9 and Table A3 of the Appendix A illustrate the mass change behavior of RAC under sulfate and chloride dry–wet cycles. Overall, in the sulfate environment, concrete mass initially increased and then sharply decreased, whereas in the chloride environment, mass exhibited a gradual upward trend. High-strength concretes displayed more stable mass evolution in both environments [31].

Under sulfate exposure (Figure 9a), RAC20 experienced a mass increase of 10.3‰ after 20 cycles, followed by significant losses of 27.3‰ and 56.8‰ at 60 and 70 cycles, respectively, indicating severe deterioration. RAC40 maintained a modest mass gain of 6.1–7.9‰ during 30–60 cycles, which declined to approximately 1.7‰ by 70 cycles. RAC60 showed the smallest variation, remaining within a 2.6–6.0‰ increase range and retaining +3.8‰ even after 70 cycles, demonstrating strong stability under sulfate attack.

In the chloride environment (Figure 9b), three groups of concrete specimens exhibited a slight but continuous mass increase. RAC20 transitioned from a slight loss of −0.13‰ at 10 cycles to a net gain of +0.35‰ at 70 cycles. RAC40 and RAC60 showed more pronounced gains, ultimately increasing by +0.53‰ and +0.65‰, respectively. This difference suggests that chloride crystallization primarily contributed to pore filling without causing significant damage to the concrete matrix, while the denser high-strength matrix enhanced the net mass gain effect.

The mass change pattern in the sulfate environment follows a typical “initial increase followed by decline”. Initially, sulfate ions react with hydration products in the pores to form ettringite and gypsum, leading to a temporary mass increase. However, as reaction products accumulate and generate expansive cracks, concrete gradually spalls, causing rapid mass loss. RAC20, with higher porosity and greater water absorption, experienced faster internal damage accumulation, resulting in the most severe mass loss. In contrast, RAC60, benefiting from a low water–binder ratio and secondary calcium silicate hydrate generated by active supplementary cementitious materials, developed a denser ITZ that effectively hindered sulfate penetration, maintaining relatively stable mass in the later cycles. In chloride exposure, crystal filling dominates, leading only to minor mass increases without significant deterioration.

#### 3.2.3. Open Porosity

**Figure 10:**
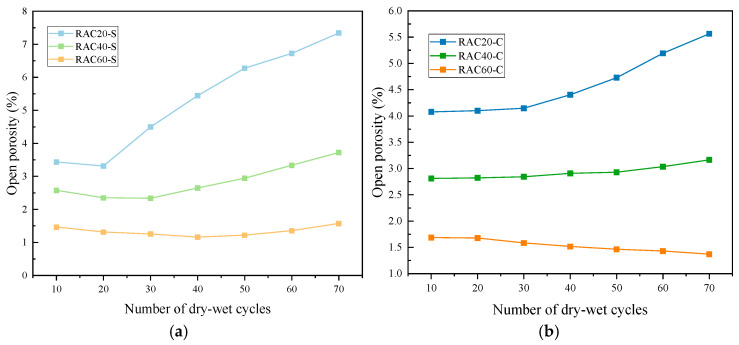
Open porosity of RAC with different strengths under (**a**) sulfate and (**b**) chloride dry–wet cycles.

The evolution of porosity for RAC with different strength levels under sulfate and chloride dry–wet cycles is presented in Figure 10 and Table A4 of the Appendix A. Overall, the sulfate environment induced a significant increase in porosity, particularly for low-strength concrete, whereas porosity changes in the chloride environment were relatively minor, with high-strength concrete even exhibiting slight densification.

As illustrated in Figure 10a, in the sulfate environment, the porosity of RAC20 initially decreased slightly during the first 20 cycles (from 3.43% to 3.31%), followed by a rapid increase, reaching 7.34% at 70 cycles—more than doubling—indicating severe internal deterioration. RAC40 maintained a relatively stable porosity (~2.35%) during the first 30 cycles and then gradually increased to 3.72%. In contrast, RAC60 showed a slight densification effect during the first 40 cycles, with porosity decreasing from 1.46% to 1.16%, and subsequently rose slowly to 1.57%. The overall fluctuation in RAC60 was much smaller than in the lower-strength groups, demonstrating its superior resistance to sulfate attack [32].

Under chloride exposure, all three RAC groups exhibited a gradual increase in porosity, as shown in Figure 10b. RAC20 increased steadily from 4.08% to 5.56%, representing an approximate 36% rise, while RAC40 rose more moderately from 2.81% to 3.16%. Notably, RAC60 exhibited a slight reduction in porosity, decreasing from 1.68% to 1.37% between 10 and 70 cycles, indicating significant densification. This suggests that the crystallization and filling effect of chloride in the pores contributes positively to the pore structure of high-strength concrete, whereas low-strength concrete, with a weaker ITZ, still experiences a gradual increase in porosity [33].

The rapid porosity increase in the sulfate environment is primarily attributed to the formation of expansive ettringite and gypsum through reactions with hydration products, which induce microcracking and continuous pore development, significantly increasing the effective porosity [34]. In contrast, in the chloride environment, crystallization predominantly fills the pores. High-strength concrete, due to its dense matrix and secondary reactions of active supplementary cementitious materials, exhibits a slight reduction in porosity.

### 3.3. Durability

#### 3.3.1. Erosion Depth

**Figure 11:**
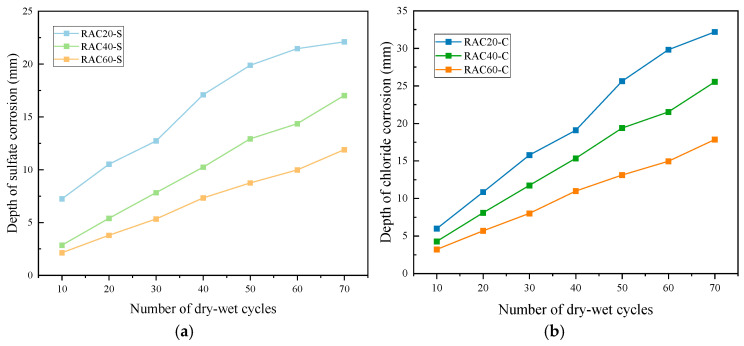
Erosion depth of RAC with different strengths under (**a**) sulfate and (**b**) chloride dry–wet cycles.

As shown in Figure 11, under both chloride and sulfate dry–wet cycles, all RAC specimens of three design strengths exhibited a progressive increase in erosion depth with the number of cycles, and the specific test data can be found in Table A5 of the Appendix A. However, the erosion rates varied significantly depending on the type of salt and the concrete strength.

For RAC20, the chloride-induced erosion depth increased from 4.46 mm at 10 cycles to 24.85 mm at 70 cycles, representing more than a fivefold increase. Sulfate attack was even more severe, with the erosion depth reaching 32.19 mm at 70 cycles—nearly 30% higher than under chloride conditions. This indicates that low-strength concrete, with higher porosity and a weaker ITZ, allows ions to penetrate more easily. RAC40 exhibited substantially lower erosion depths compared to RAC20. Under chloride exposure, the depth increased from 3.36 mm at 10 cycles to 20.22 mm at 70 cycles, while under sulfate exposure it rose from 4.26 mm to 25.53 mm. Compared with RAC20, the 70-cycle erosion depths under chloride and sulfate conditions were reduced by 18.6% and 20.7%, respectively, demonstrating the beneficial effect of improved matrix density in moderating ion penetration. RAC60 showed the best erosion resistance. Under chloride conditions, the depth increased modestly from 2.45 mm at 10 cycles to 14.65 mm at 70 cycles, while under sulfate conditions it rose from 3.20 mm to 17.84 mm. Compared with RAC20, the erosion depths for RAC60 at 70 cycles decreased by 41.0% and 44.6% under chloride and sulfate conditions, respectively. This superior performance is attributed to the low water–cement ratio and dense microstructure of high-strength concrete, which effectively hinder the penetration and diffusion of chloride and sulfate ions [35].

Overall, sulfate attack induced greater erosion depths than chloride attack, particularly in low-strength concrete. This is attributed to the chemical reactions of sulfate ions with hydration products, forming expansive compounds such as ettringite and gypsum, which promote microcrack propagation and pore connectivity, thereby accelerating the erosion process. In contrast, chloride attack mainly involves physical penetration and crystallization pressure, resulting in a relatively slower degradation rate.

#### 3.3.2. Erosion Content

**Figure 12:**
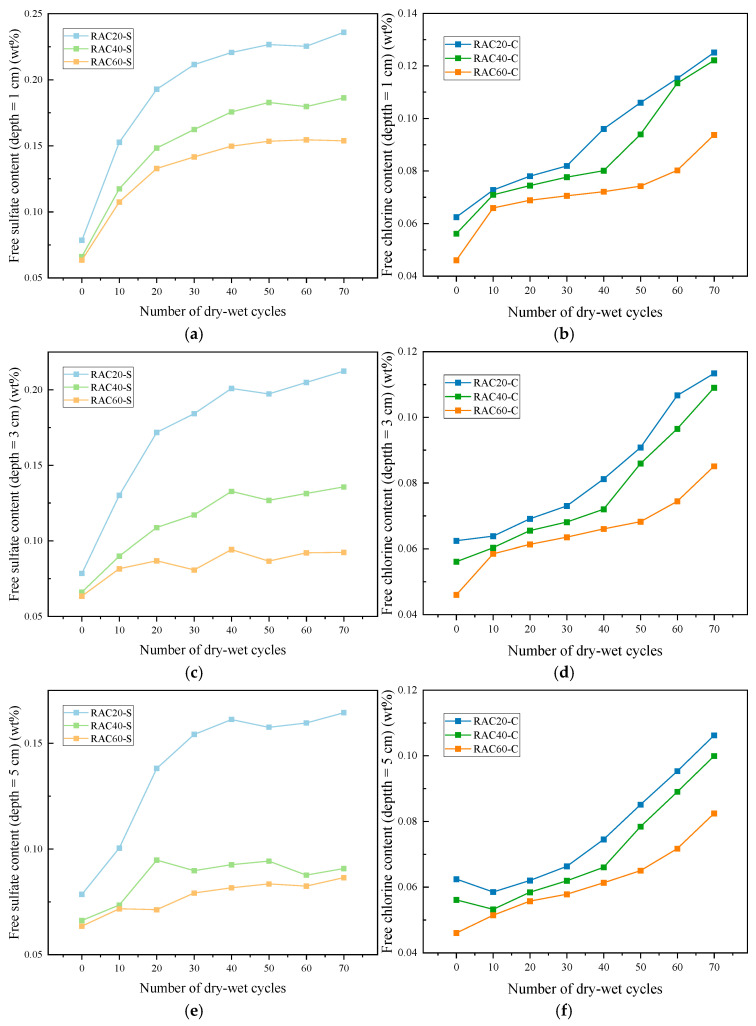
(**a**–**c**) Chloride and (**d**–**f**) sulfate ion content in RFAC of different strengths under dry–wet cycling.

Figure 12 and Table A6 of the Appendix A illustrate the variation of sulfide and chloride ion contents in recycled concrete samples with different design strengths under dry–wet cycling. Overall, both sulfide and chloride ions exhibit progressive accumulation with increasing cycles, but the enrichment rate and distribution characteristics vary significantly with concrete strength.

For RAC20, the sulfide content at 1 cm depth increased from 0.0785 wt% initially to 0.2359 wt% after 70 days, representing an approximate 200% rise; correspondingly, the chloride content nearly doubled from 0.0624 wt% to 0.1251 wt%. As depth increased, the accumulation rate of both ions decreased, with sulfide and chloride contents at 5 cm depth after 70 days reaching 0.1645 wt% and 0.1062 wt%, respectively, significantly lower than the surface. This indicates that the high porosity and weak ITZ of low-strength RAC20 facilitate ion migration and rapid surface enrichment, resulting in pronounced superficial degradation [36].

For RAC40, the sulfide content at 1 cm depth reached 0.1863 wt% after 70 days, increasing by approximately 182% from the initial value, while chloride content rose from 0.0561 wt% to 0.1221 wt%. At 3 cm and 5 cm depths, ion accumulation was considerably lower; after 70 days, sulfide and chloride contents at 5 cm depth were 0.0907 wt% and 0.0999 wt%, respectively. Compared with RAC20, the reduced ion migration gradient in RAC40 reflects the more compact microstructure, which partially impedes ion penetration. RAC60 exhibited the least ion accumulation. At 1 cm depth, sulfide content increased from 0.0634 wt% to 0.1538 wt% (approximately 142% increase), and chloride content rose from 0.046 wt% to 0.0937 wt%, both significantly lower than those of RAC20 and RAC40. At 5 cm depth, sulfide and chloride contents after 70 days were 0.0864 wt% and 0.0824 wt%, roughly 50–60% of surface values. This demonstrates that the low water–cement ratio and dense ITZ of high-strength RAC60 create an effective barrier layer, inhibiting deep migration and accumulation of external ions.

From the perspective of degradation mechanisms, sulfate attack primarily involves the reaction of external sulfate ions with hydration products to form gypsum and ettringite, accompanied by volumetric expansion and microcrack propagation, which accelerates surface degradation and promotes superficial ion accumulation [35]. In contrast, chloride attack mainly occurs through the penetration of chloride ions, which react with calcium hydroxide and aluminates in the hydration products to form calcium chloroaluminate, thereby compromising pore integrity and reducing the effectiveness of the concrete cover in protecting steel reinforcement [37]. The alternating action of these two mechanisms under dry–wet cycling gradually enlarges the concrete’s pores and accelerates structural deterioration.

### 3.4. Microscopic Properties

#### 3.4.1. XRD Analysis

**Figure 13:**
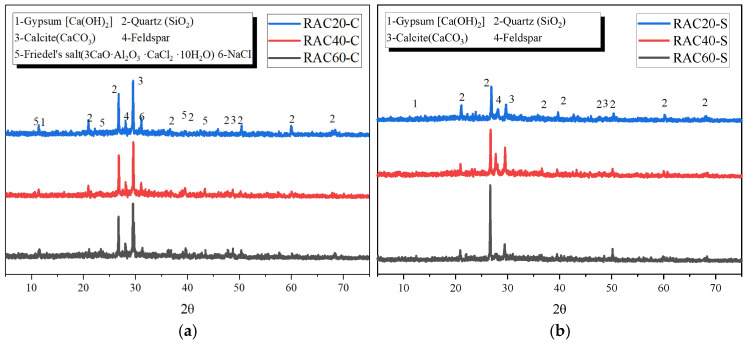
XRD patterns of RAC with different strengths under (**a**) chloride and (**b**) sulfate dry–wet cycles.

As shown in Figure 13a, under chloride dry–wet cycling, the XRD patterns of RAC20, RAC40, and RAC60 exhibited characteristic peaks of portlandite, silica, calcite, feldspar, and Friedel’s salt, with the NaCl peaks being particularly prominent. This indicates significant penetration of chloride ions into the concrete. As the cycles progressed, the intensity of Friedel’s salt peaks gradually increased, suggesting that some chloride ions reacted with cement hydration products to form Friedel’s salt. While this reaction partially immobilizes chloride ions, the volumetric expansion of the salt products accelerates pore enlargement and microcrack development, thereby promoting concrete degradation. Among the different strengths, RAC20 showed the most pronounced changes in diffraction peaks, reflecting its higher porosity and easier chloride ion migration, whereas RAC60, with its dense microstructure, exhibited chloride accumulation mainly near the surface and relatively fewer internal reaction products, resulting in lower degradation.

In contrast, under sulfate dry–wet cycling (Figure 13b), the XRD patterns revealed peaks corresponding to ettringite and gypsum. Extensive sulfate ion penetration into the pores reacted with hydration products, forming additional ettringite and gypsum. Due to their significant expansive characteristics, this induced further cracking and increased porosity. Among the different strengths, RAC20 showed a marked decrease in the intensity of calcium silicate hydrate peaks after sulfate cycling, indicating that its low density facilitated sulfate penetration and reaction with calcium silicate hydrate. In contrast, RAC60’s dense microstructure effectively slowed sulfate ingress, limiting the internal formation of expansive products, although ettringite formation was still observable near the surface.

Overall, the comparison indicates that chloride attack is characterized primarily by chloride penetration and Friedel’s salt formation, whereas sulfate attack manifests as the formation of ettringite and gypsum and their associated expansion. The effects of chloride are more pronounced in low-strength concrete due to the abundance of ion migration pathways and rapid degradation, while sulfate attack induces severe calcium silicate hydrate reaction and pore expansion in low-strength concrete. High-strength concrete, with its denser structure, demonstrates significantly higher resistance. These results highlight that concrete strength and matrix density are key factors determining its ability to resist different types of salt-induced deterioration.

#### 3.4.2. SEM Analysis

**Figure 14:**
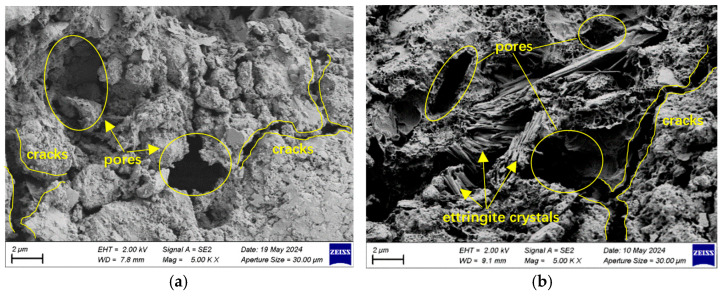

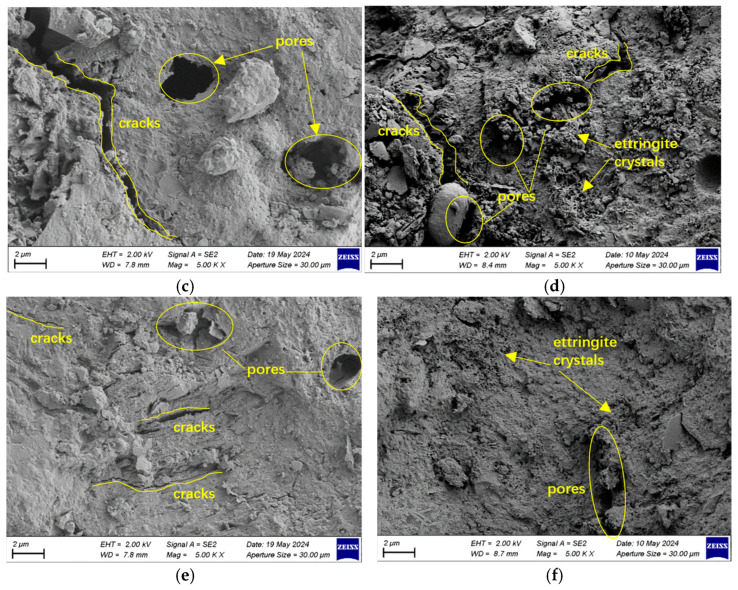
SEM morphologies of RAC under dry–wet cycles: (**a**,**b**) RAC20; (**c**,**d**) RAC40; (**e**,**f**) RAC60 ((**a**,**c**,**e**) under the chloride exposure, (**b**,**d**,**f**) under the sulfate exposure).

Figure 14a,c,e presents SEM observations of RAC20, RAC40, and RAC60 after 70 cycles of chloride exposure. The SEM images show that chloride ions penetrated into the concrete and reacted with hydration products to form Friedel’s salt and expansive complex salts [38]. This reaction consumed a considerable amount of hydroxide ions, lowering the pore solution pH and causing partial decomposition of calcium silicate hydrate gel, further deteriorating the pore structure. For RAC20 and RAC40, the high content of adhered mortar in the recycled aggregates and the weak ITZ led to pronounced internal cracks and pore development, accelerating chloride diffusion and penetration and resulting in a more porous structure. In contrast, RAC60’s dense microstructure effectively hindered ion penetration, resulting in relatively minor surface damage. Overall, following chloride salt exposure, the surface of RAC typically exhibited a rough and porous morphology, accompanied by discontinuous cracking. Among them, RAC20 displayed wide and extensive cracks, the highest pore density, and a loose surface texture with abundant hydration product particles distributed throughout. In contrast, RAC40 exhibited relatively narrower and shorter cracks, fewer pores, and a noticeable reduction in surface hydration products. RAC60, however, showed the narrowest cracks, the lowest porosity, and a smoother surface, indicating a significantly denser microstructure. These findings demonstrate that increasing the designed strength effectively refines the pore structure and enhances the microstructural compactness of RAC.

SEM results after sulfate dry–wet cycling are presented in Figure 14b,d,f. For RAC20, numerous ettringite crystals were observed along cracks and surface regions, indicating deep sulfate penetration and expansive reactions with hydration products. Due to the initially high porosity and pre-existing cracks in RAC20, sulfate ions rapidly infiltrated through these pathways, continuously reacting with hydration products to generate new cracks and accelerate structural degradation, leading to a severely weakened and porous microstructure and a substantial loss of effective load-bearing capacity. In contrast, RAC40 exhibited a relatively dense overall structure, with limited ettringite deposition around cracks and markedly reduced surface cracking and porosity. These observations suggest only initial sulfate ingress and limited reaction with hydration products, leading to relatively minor structural damage. For RAC60, SEM images showed smooth cross-sections, with sparse ettringite formation near pores and only a few isolated surface pores; no significant crack propagation was detected. These findings indicate that sulfate penetration in RAC60 was limited, internal formation of sulfates was minimal, and the results are consistent with macroscopic durability tests, further confirming the excellent sulfate resistance of high-strength RAC.

## 4. Conclusions

This study systematically evaluated the mechanical performance, durability, and microstructural evolution of RAC with different design strengths (20, 40, and 60 MPa) under chloride and sulfate dry–wet cycles. The main findings are summarized as follows:

(1) All RAC groups with three design strengths achieved their target compressive strengths, and a clear negative correlation was observed between open porosity and strength. The open porosities of RAC20, RAC40, and RAC60 were 4.03%, 2.35%, and 1.70%, respectively, indicating that the strength enhancement is closely associated with matrix densification.
(2) Under sulfate exposure, the compressive strength of RAC20 decreased by 60% after 70 cycles, showing severe degradation. RAC40 experienced an 18.0% loss, whereas RAC60 maintained a net gain of +2.5%, demonstrating strong resistance to sulfate attack. In chloride environments, strength reductions were milder: RAC20 lost 30.7%, RAC40 lost 6.9%, and RAC60 still exhibited a +6.6% increase. These results indicate that high-strength matrices effectively resist aggressive ion penetration.
(3) In sulfate environments, the mass of RAC20 initially increased but then sharply declined, with a final loss of 56.8‰, whereas RAC60 consistently retained a net gain of +3.8‰. Similar trends were observed in porosity evolution: the porosity of RAC20 more than doubled to 7.34%, while RAC60’s porosity decreased to 1.16% during the first 40 cycles and only slightly increased to 1.57% thereafter, highlighting its remarkable corrosion resistance. Under chloride exposure, all three groups showed slight increases in mass or porosity, or even densification, with high-strength concrete exhibiting the most pronounced improvement.
(4) After 70 cycles, the chloride and sulfate penetration depths in RAC20 reached 24.85 mm and 32.19 mm, respectively, whereas RAC60 showed significantly lower values of 14.65 mm and 17.84 mm, corresponding to reductions of 41.0% and 44.6%. Ion content measurements revealed that low-strength concretes accumulated significantly more chloride ions and sulfates in the shallow layer (0–30 mm) than high-strength counterparts, indicating that porosity and ITZ densification govern ion diffusion rates and distribution patterns.
(5) SEM and XRD analyses indicate that, under sulfate exposure, the primary degradation mechanism is the expansive formation of ettringite and gypsum, which leads to increased porosity and propagation of microcracks. In contrast, under chloride exposure, degradation is mainly dominated by crystal filling and localized chemical reactions, while the formation of calcium silicate hydrate in high-strength concrete effectively suppresses pore expansion and mitigates deterioration.

This study investigated the deterioration mechanisms of dry–wet cycling performance in RAC of different strength grades under individual exposure to chloride and sulfate salts. However, in practical erosion environments, chlorides and sulfates often coexist and typically act synergistically on the material in a coupled manner. Therefore, the performance evolution behavior and deterioration mechanisms of recycled concrete across strength grades under coupled chloride–sulfate dry–wet cycling conditions require further in-depth investigation, representing a key direction for future research.

## Figures and Tables

**Table 1:** The chemical compositions and properties of cementitious materials.

		P.O 32.5 Cement	P.O 42.5 Cement	Fly Ash	Silica Fume
Elemental proportion (wt%)	CaO	60.19	59.22	3.81	0.85
	SiO_2_	19.21	18.55	52.53	88.24
	Al_2_O_3_	8.55	9.02	28.33	0.77
	Fe_2_O_3_	3.68	4.55	3.7	0.85
	MgO	1.27	1.36	1.16	0.94
	MnO	0.14	0.12	0.21	<0.01
	K_2_O	0.71	0.63	1.70	1.28
	TiO_2_	0.24	0.20	0.94	<0.01
	SO_3_	2.08	2.03	1.88	<0.01
	Cl	0.04	0.03	<0.01	<0.01
Loss on ignition (wt%)		3.20	2.57	1.83	1.79
Specific surface area (m^2^/kg)		380	369	430	23,000
Apparent density (kg/m^3^)		3060	3152	2524	2721
Bulk density (kg/m^3^)		1400	1447	1120	270

**Table 2:** Physical and mechanical properties of aggregates.

	Apparent Density (kg/m^3^)	Water Absorption (wt%)	Crushing Index (wt%)
Natural coarse aggregate	2687	0.4	4.3
Recycled fine aggregate	2394	8.8	25.6

**Table 3:** Mix proportions of concrete (kg/m^3^).

Component	RAC20	RAC40	RAC60
Natural coarse aggregate	1017	1081	1180
Recycled fine aggregate	649	591	503
Cement	421	429	370
Fly ash	0	50	114
Silica fume	0	25	85
Water	152	134	102
Superplasticizer	0.63	0.75	0.85

**Table 4:** The number of specimens and their purpose.

Specimens				Specimen Purpose
RAC20	RAC40	RAC60	Total	
6	6	6	18	Compressive strength at 7 and 28 days of curing.
36	36	36	108	Compressive strength, free chloride ion content, and chloride penetration depth during chloride salt dry–wet cycles.
3	3	3	9	Open porosity variation and mass change during chloride salt dry–wet cycles.
36	36	36	108	Compressive strength, free sulfate ion content, and sulfate penetration depth during sulfate dry–wet cycles.
3	3	3	9	Open porosity variation and mass change during sulfate dry–wet cycles.
36	36	36	108	Compressive strength, free chloride and sulfate ion content, and chloride and sulfate penetration depth during compound salt dry–wet cycles.
3	3	3	9	Open porosity variation and mass change during compound salt dry–wet cycles.
4	4	4	16	For backup.

## Data Availability

The original contributions presented in this study are included in the article. Further inquiries can be directed to the corresponding authors.

## Appendix A

**Table A1:** Initial mechanical and porosity properties.

Concrete	Compressive Strength /MPa	Flexural Strength /MPa	Splitting Tensile Strength /MPa	Open Porosity /(vol%)
RAC20	20.2 (1.3)	2.6 (0.3)	1.9 (0.4)	4.03 (0.07)
RAC40	47.1 (2.1)	5.9 (0.6)	3.9 (0.3)	2.82 (0.14)
RAC60	62.7 (2.2)	7.8 (0.7)	5.1 (0.5)	1.70 (0.06)

Note: The numbers in parentheses indicate the measurement errors.

**Table A2:** Compressive strength variation in RACs under chloride and sulfate dry–wet cycles (%).

Dry–Wet Cycles	10	20	30	40	50	60	70
RAC20-S	11.79 (0.9)	14.15 (1.0)	−6.43 (0.4)	−20.30 (1.3)	−38.81 (2.8)	−50.30 (3.6)	−60.09 (4.5)
RAC40-S	4.88 (0.4)	9.34 (0.7)	9.55 (0.6)	3.40 (0.3)	−2.55 (0.1)	−10.40 (0.9)	−18.05 (1.1)
RAC60-S	4.70 (0.3)	7.72 (0.4)	8.89 (0.5)	10.74 (0.9)	9.56 (0.4)	6.88 (0.5)	2.52 (0.4)
RAC20-C	−0.93 (0.2)	−1.40 (0.2)	−2.33 (0.1)	−7.44 (0.5)	−13.95 (1.0)	−23.26 (1.8)	−30.70(2.3)
RAC40-C	0.22 (0.1)	0.00 (0.1)	−0.43 (0.1)	−1.72 (0.1)	−2.15 (0.2)	−4.30 (0.3)	−6.88 (0.4)
RAC60-C	0.34 (0.2)	0.51 (0.1)	2.38 (0.2)	3.74 (0.2)	4.75 (0.3)	5.43 (0.4)	6.62 (0.4)

Note: The numbers in parentheses indicate the measurement errors.

**Table A3:** Mass change behavior of RAC under sulfate and chloride dry–wet cycles (%).

Dry–Wet Cycles	10	20	30	40	50	60	70
RAC20-S	6.71 (0.5)	10.29 (0.7)	7.16 (0.6)	0.89 (0.1)	−4.92 (0.3)	−20.29 (1.5)	−46.82 (3.6)
RAC40-S	3.90 (0.2)	6.07 (0.4)	7.81 (0.7)	7.38 (0.6)	6.51 (0.5)	6.94 (0.5)	1.74 (0.2)
RAC60-S	2.55 (0.2)	3.83 (0.3)	4.68 (0.4)	5.11 (0.4)	5.96 (0.4)	4.68 (0.4)	3.83 (0.3)
RAC20-C	−0.13 (0.1)	−0.18 (0.1)	−0.21 (0.1)	−0.04 (0.0)	0.13 (0.1)	0.21 (0.1)	0.35 (0.1)
RAC40-C	−0.11 (0.1)	−0.16 (0.1)	−0.19 (0.1)	0.10 (0.1)	0.29 (0.1)	0.37 (0.1)	0.53 (0.1)
RAC60-C	−0.09 (0.0)	−0.14 (0.1)	−0.17 (0.1)	0.19 (0.1)	0.38 (0.1)	0.45 (0.1)	0.65 (0.2)

Note: The numbers in parentheses indicate the measurement errors.

**Table A4:** Evolution of porosity for RA under sulfate and chloride dry–wet cycles (%).

Dry–Wet Cycles	10	20	30	40	50	60	70
RAC20-S	3.43 (0.21)	3.31 (0.23)	4.49 (0.32)	5.44 (0.44)	6.28 (0.46)	6.72 (0.42)	7.34 (0.52)
RAC40-S	2.57 (0.11)	2.35 (0.16)	2.33 (0.25)	2.64 (0.21)	2.94 (0.18)	3.33 (0.24)	3.72 (0.19)
RAC60-S	1.46 (0.12)	1.31 (0.08)	1.25 (0.32)	1.16 (0.08)	1.22 (0.10)	1.35 (0.09)	1.57 (0.12)
RAC20-C	4.08 (0.32)	4.10 (0.28)	4.15 (0.12)	4.40 (0.27)	4.73 (0.28)	5.19 (0.42)	5.56 (0.38)
RAC40-C	2.81 (0.15)	2.82 (0.16)	2.84 (0.26)	2.91 (0.11)	2.93 (0.18)	3.03 (0.22)	3.16 (0.24)
RAC60-C	1.68 (0.10)	1.67 (0.11)	1.58 (0.12)	1.51 (0.08)	1.46 (0.10)	1.43 (0.12)	1.37 (0.09)

Note: The numbers in parentheses indicate the measurement errors.

**Table A5:** Evolution of erosion depth of RAC under sulfate and chloride dry–wet cycles (mm).

Dry–Wet Cycles	10	20	30	40	50	60	70
RAC20-S	7.23 (0.47)	10.52 (1.04)	12.72 (0.89)	17.09 (1.30)	19.88 (1.28)	21.46 (1.26)	22.10 (1.32)
RAC40-S	2.84 (0.30)	5.39 (0.73)	7.82 (0.62)	10.23 (0.88)	12.92 (1.03)	14.35 (1.31)	17.02 (1.12)
RAC60-S	2.13 (0.21)	3.78 (0.36)	5.33 (0.46)	7.32 (0.81)	8.74 (0.97)	9.97 (1.02)	11.89 (1.06)
RAC20-C	5.97 (0.42)	10.85 (0.95)	15.78 (1.24)	19.08 (1.22)	25.64 (1.33)	29.82 (2.04)	32.19 (1.66)
RAC40-C	4.26 (0.38)	8.09 (0.67)	11.73 (1.01)	15.35 (1.12)	19.38 (0.96)	21.53 (1.23)	25.53 (1.44)
RAC60-C	3.20 (0.31)	5.67 (0.46)	8.00 (0.66)	10.98 (0.91)	13.11 (0.84)	14.96 (1.05)	17.84 (0.85)

Note: The numbers in parentheses indicate the measurement errors.

**Table A6:** Evolution of erosion ion content under sulfate and chloride dry–wet cycles (mm).

	Cycles	10	20	30	40	50	60	70
10 mm	RAC20-S	0.079 (0.008)	0.153 (0.011)	0.193 (0.009)	0.212 (0.013)	0.221 (0.007)	0.227 (0.010)	0.225 (0.008)
	RAC40-S	0.066 (0.006)	0.117 (0.010)	0.148 (0.008)	0.162 (0.010)	0.176 (0.011)	0.183 (0.009)	0.180 (0.009)
	RAC60-S	0.063 (0.006)	0.107 (0.008)	0.133 (0.005)	0.142 (0.009)	0.150 (0.007)	0.153 (0.006)	0.155 (0.010)
	RAC20-C	0.062 (0.005)	0.073 (0.009)	0.078 (0.006)	0.082 (0.006)	0.096 (0.009)	0.106 (0.005)	0.115 (0.009)
	RAC40-C	0.056 (0.006)	0.071 (0.006)	0.074 (0.007)	0.078 (0.007)	0.080 (0.005)	0.094 (0.007)	0.113 (0.006)
	RAC60-C	0.046 (0.005)	0.066 (0.007)	0.069 (0.008)	0.071 (0.005)	0.072 (0.007)	0.074 (0.008)	0.080 (0.005)
30 mm	RAC20-S	0.079 (0.005)	0.130 (0.007)	0.172 (0.010)	0.184 (0.007)	0.201 (0.011)	0.197 (0.008)	0.205 (0.009)
	RAC40-S	0.066 (0.006)	0.090 (0.006)	0.109 (0.005)	0.117 (0.008)	0.133 (0.007)	0.127 (0.011)	0.131 (0.005)
	RAC60-S	0.063 (0.005)	0.082 (0.008)	0.087 (0.007)	0.081 (0.006)	0.094 (0.008)	0.087 (0.007)	0.092 (0.006)
	RAC20-C	0.062 (0.008)	0.064 (0.009)	0.069 (0.009)	0.073 (0.005)	0.081 (0.005)	0.091 (0.005)	0.107 (0.007)
	RAC40-C	0.056 (0.005)	0.060 (0.005)	0.066 (0.005)	0.068 (0.007)	0.072 (0.009)	0.086 (0.006)	0.097 (0.009)
	RAC60-C	0.046 (0.007)	0.059 (0.007)	0.061 (0.008)	0.064 (0.006)	0.066 (0.005)	0.068 (0.008)	0.074 (0.005)
50 mm	RAC20-S	0.079 (0.006)	0.100 (0.009)	0.138 (0.008)	0.154 (0.011)	0.161 (0.008)	0.158 (0.012)	0.160 (0.010)
	RAC40-S	0.066 (0.005)	0.073 (0.007)	0.095 (0.009)	0.090 (0.010)	0.093 (0.009)	0.094 (0.010)	0.088 (0.008)
	RAC60-S	0.063 (0.007)	0.072 (0.005)	0.071 (0.006)	0.079 (0.007)	0.082 (0.007)	0.083 (0.007)	0.082 (0.008)
	RAC20-C	0.062 (0.007)	0.059 (0.007)	0.062 (0.007)	0.066 (0.008)	0.075 (0.006)	0.085 (0.006)	0.095 (0.005)
	RAC40-C	0.056 (0.008)	0.053 (0.006)	0.058 (0.005)	0.062 (0.007)	0.066 (0.005)	0.078 (0.008)	0.089 (0.007)
	RAC60-C	0.046 (0.005)	0.051 (0.006)	0.056 (0.007)	0.058 (0.005)	0.061 (0.006)	0.065 (0.007)	0.072 (0.005)

Note: The numbers in parentheses indicate the measurement errors.
